# Development and validation of a clinical prediction model for sepsis-induced cardiomyopathy

**DOI:** 10.3389/fcvm.2026.1788162

**Published:** 2026-07-17

**Authors:** Tenghao Shao, Dan Su, Jinwen Zhang, Wenchao Kan, Yingxin Wang, Jiaqian Wu, Nan Zhang, Na Cui, Hongwei Zhang

**Affiliations:** 1 Department of Intensive Care Unit, Affiliated Hospital of Hebei University, Baoding City, China; 2 Department of Medical Clinic, Tangshan College, Tangshan City, China

**Keywords:** critical care, development, propensity score matching, risk prediction model, sepsis-induced cardiomyopathy, validation

## Abstract

**Objective:**

The objective of this study was to develop and validate a clinically applicable risk-prediction model for sepsis-induced cardiomyopathy (SICM) and to evaluate its predictive performance comprehensively.

**Methods:**

A retrospective cohort study was conducted using clinical data from patients with sepsis, obtained from the Medical Information Mart for Intensive Care IV database. Propensity score matching was applied to minimize confounding and achieve balance in baseline characteristics between groups. Candidate predictors were initially screened using univariate analysis, and feature selection was performed using the least absolute shrinkage and selection operator regression method. A multivariate logistic regression model was subsequently developed and externally validated with an independent dataset.

**Results:**

The prediction model was derived from 956 patients and externally validated in a cohort of 104 patients. Five independent predictors were retained in the final model: serum phosphate concentration, neutrophil percentage, troponin concentration, heart rate, and Charlson Comorbidity Index. The model demonstrated strong discriminatory ability, with C-statistic values of 0.80 in the derivation cohort, 0.79 in the internal validation cohort, and 0.76 in the external validation cohort.

**Conclusion:**

The validated prediction model provides accurate estimation of SICM risk. Based on routinely available clinical variables, the model has potential utility for early risk stratification and individualized management of patients with sepsis, supporting improved clinical outcomes in those at elevated risk of SICM.

## 1 Introduction

Sepsis-induced cardiomyopathy (SICM) is characterized by acute cardiac dysfunction occurring in patients with sepsis and is mediated through mechanisms such as systemic inflammation, microcirculatory impairment, and metabolic dysregulation. This condition has been associated with substantially increased risks of multi-organ failure and mortality (1, 2). Early detection and intervention may enhance hemodynamic support and improve patient outcomes. However, accurate diagnosis of SICM remains challenging due to its nonspecific clinical manifestations and the limited sensitivity and specificity of currently available diagnostic modalities, including echocardiography and circulating biomarkers (3, 4).

Prognostic scoring systems such as the Acute Physiology and Chronic Health Evaluation II (APACHE II) and the Sequential Organ Failure Assessment (SOFA) are widely used in critical care settings; however, these tools were not designed specifically for SICM risk prediction and show limited accuracy in stratifying affected patients. Several predictive models for SICM have been proposed, yet many are constrained by limitations, including inadequate selection of predictors, limited clinical applicability, and lack of external validation (5, 6).

To address these gaps, the present study was conducted to develop and validate a prediction model tailored for SICM risk estimation, based on objectively measured clinical and laboratory parameters obtained early after hospital admission. The model was systematically evaluated for discrimination, calibration, and potential clinical utility, with the aim of supporting early risk stratification and informing individualized treatment strategies.

## 2 Methods

### 2.1 Data source

Data were obtained from the Medical Information Mart for Intensive Care IV (MIMIC-IV, version 3.0) database, a publicly accessible critical care database containing detailed clinical records of patients admitted to the intensive care units at Beth Israel Deaconess Medical Center (Boston, MA, USA) between 2008 and 2022. A total of 956 patients who met the predefined inclusion criteria were included into the derivation cohort.

Access to the database was granted following completion of the Collaborative Institutional Training Initiative (CITI) program in Human Research Protection (Certification No. 62019383). The study protocol received approval from the institutional review boards of the Massachusetts Institute of Technology and Beth Israel Deaconess Medical Center, with a waiver of informed consent granted due to the de-identified nature of the data. Data extraction was conducted using Structured Query Language (SQL) via the Google BigQuery interface.

For external validation, an independent cohort of 104 patients with sepsis was retrospectively identified from the Intensive Care Unit of the Affiliated Hospital of Hebei University, China, between January 2022 and December 2024. The validation study adhered to the Declaration of Helsinki and was approved by the Ethics Committee of the Affiliated Hospital of Hebei University (Approval No.: HDFYLL-IIT-2025-081), which also waived the requirement for informed consent. The sample size was determined by the availability of eligible patients in the MIMIC-IV database.

### 2.2 Patient selection criteria

**Figure 1:**
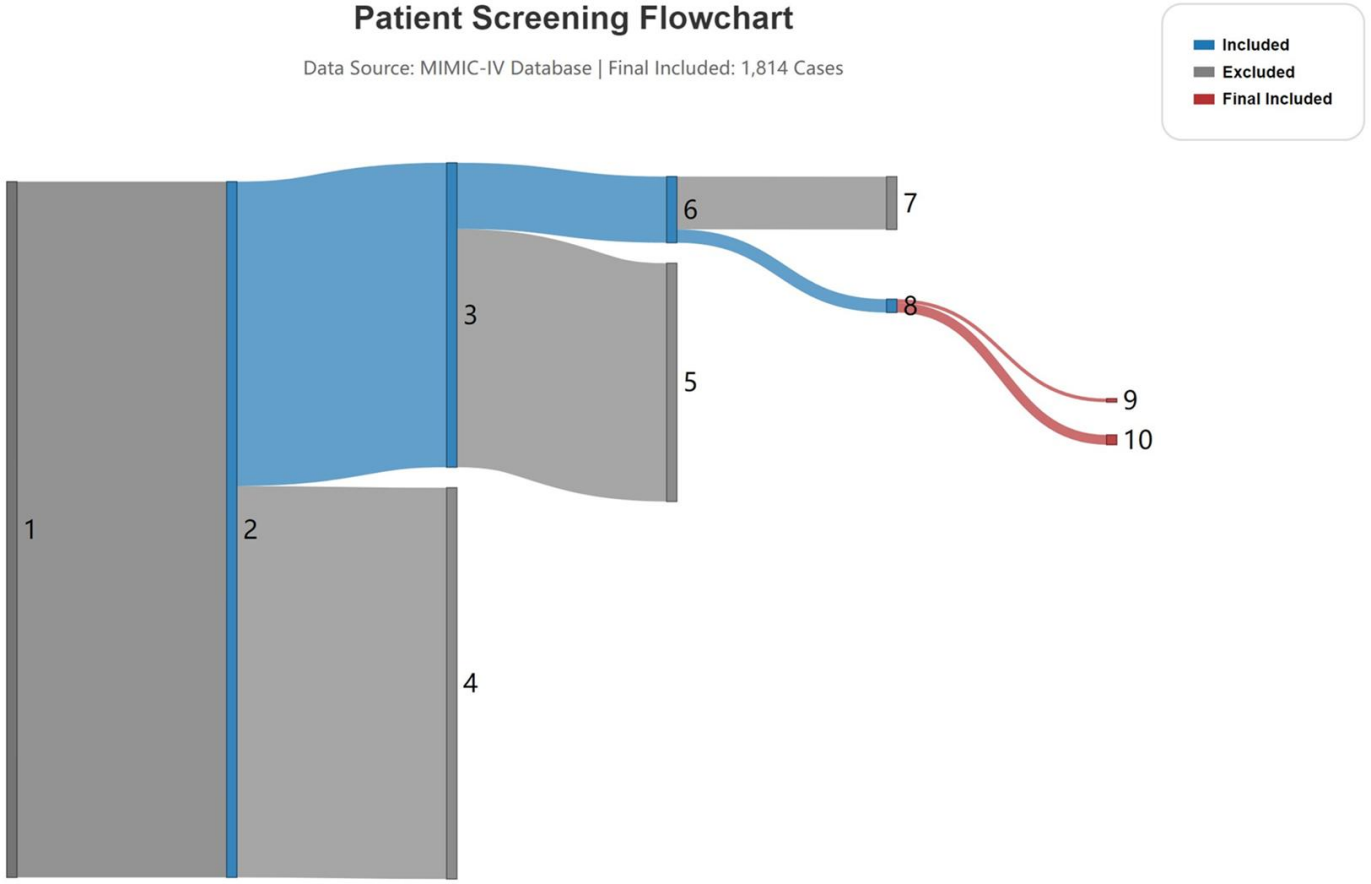
Flowchart of patient selection. This diagram illustrates the inclusion and exclusion process for patients enrolled in the SICM study.1: Total ICU Admissions (94,458), 2: Age ≥18 years (94,458), 3: Meet Sepsis Criteria (41,295), 4: Not Meet Sepsis Criteria (53,163), 5: Missing Data (32,318), 6: Complete Data (8,977), 7: CAD/Cardiomyopathy (7,163). 8: No CAD/Cardiomyopathy (1,814), 9: EF < 50% (482), 10: EF ≥ 50% (1,332).

Patients were eligible for inclusion if they met the Sepsis-3.0 diagnostic criteria (7). Exclusion criteria included age <18 years, incomplete critical baseline information, indeterminate outcome documentation, or a prior history of coronary artery disease or cardiomyopathy. For patients with multiple ICU admissions, only data from the first admission were considered (Figure 1).

### 2.3 Diagnostic criteria and study groups

SICM was defined in the present study based on established literature (8, 9) as a left ventricular ejection fraction (LVEF) < 50% measured during hospitalization in septic patients without pre-existing coronary artery disease or cardiomyopathy. Although this pragmatic definition is commonly adopted in retrospective database analyses, it fails to cover the full spectrum of SICM phenotypes, including isolated right ventricular or diastolic dysfunction—nor does it account for the potential influence of dynamic loading conditions (e.g., fluid status, vasopressor use) on LVEF measurements. To ensure clinical reproducibility and feasibility, LVEF assessments were conducted following routine clinical protocols, with no standardized adjustments for loading conditions.

For the present study, the first LVEF measurement recorded within 72 h of ICU admission (or within 24 h of meeting Sepsis-3.0 criteria) was extracted from the MIMIC-IV database to standardize timing as much as feasible given the retrospective design. Echocardiographic examinations were performed as part of routine clinical care; therefore, standardized imaging protocols, blinding, or central re-reading were not implemented. LVEF values were retrieved from clinical reports, which at the source institution (Beth Israel Deaconess Medical Center) are typically quantified using the Simpson's biplane method or visual estimation by board-certified cardiologists. Due to the retrospective design, formal interobserver variability could not be assessed.

Based on this definition, patients were divided into two groups: the non-SICM group (sepsis patients without SICM) and the SICM group (those who developed SICM).

### 2.4 Variable extraction

Variables were extracted from relevant literature and clinical records and were categorized as follows:

1. **Demographic characteristics:** age, gender, height, and weight.
2. **Initial laboratory parameters** (measured within the first 24 h of ICU admission):
  **Electrolytes and minerals:** calcium (calcium_icu24h), chloride (chloride_icu24h), magnesium (magnesium_icu24h), potassium (potassium_icu24h), sodium (sodium_icu24h), and phosphate (phosphate_icu24h).
  **Liver function and protein markers:** albumin (albumin_icu24h), alkaline phosphatase (alkaline_icu24h), alanine aminotransferase (alt_icu24h), aspartate aminotransferase (ast_icu24h), and total bilirubin (tbilirubin_icu24h).
  **Hematologic parameters:** lymphocyte percentage (lymph_icu24h), neutrophil percentage (neu_icu24h), hematocrit (hematocrit_icu24h), hemoglobin (hemoglobin_icu24h), mean corpuscular hemoglobin (mch_icu24h), mean corpuscular volume (mcv_icu24h), mean corpuscular hemoglobin concentration (mchc), platelet count (platelet_icu24h), red blood cell count (rbc_icu24h), red cell distribution width (rdw_icu24h), and white blood cell count (wbc_icu24h).
  **Coagulation indices:** activated partial thromboplastin time (aptt_icu24h), international normalized ratio (inr_icu24h), and prothrombin time (pt_icu24h).
  **Renal and cardiac biomarkers:** creatinine (creatinine_icu24h), creatine kinase-MB (ckmb_icu24h), and troponin (troponin_icu24h).
  **Metabolic and acid–base indicators:** glucose (glu_icu24h), base excess (base_icu24h), lactate (lac_icu24h), partial pressure of carbon dioxide (pco2_icu24h), pH (ph_icu24h), and partial pressure of oxygen (po2_icu24h).
3. **Initial vital signs** (measured within the first 24 h of admission): heart rate (heartrate_icu24h), mean arterial pressure (mbp_icu24h), respiratory rate (resprate_icu24h), oxygen saturation (spo2_icu24h), and body temperature (temperature_icu24h).
4. **Clinical scores** (assessed within the first 24 h of admission): Acute Physiology and Chronic Health Evaluation III (APACHE III; apsiii), Charlson Comorbidity Index (charlson), Glasgow Coma Scale (init_gcs), and Sequential Organ Failure Assessment score [SAPS II (sapsii), SOFA score; init_sofa].
5. **Interventions within the first 24 h of admission:** continuous renal replacement therapy (CRRT), antibiotics (antibiotics), analgesic and sedative medications (analgesics), diuretics (furosemide), vasoactive drugs (vasoactive), and invasive mechanical ventilation (invasive vent).
6. **Comorbid conditions:** diabetes mellitus (diabetes), chronic obstructive pulmonary disease (COPD), asthma (asthma), and hypertension (hypertension).

### 2.5 Handling of missing data

In the derivation cohort, a substantial proportion of variables contained missing values. Variables with a missing rate >40% were excluded from subsequent analyses (Supplementary Table S1). This threshold was adopted in accordance with the standard practice for large-scale database studies using the MIMIC-IV dataset to balance retention of clinically relevant predictors against the risk of bias introduced by imputing variables with very high rates of missingness. For the remaining variables, multiple imputation was performed using the Monte Carlo method (fully conditional specification) under the missing at random (MAR) assumption, i.e., the probability of missingness depends on observed data but not on unobserved values. Five complete imputed datasets were generated. All subsequent statistical analyses were conducted using these imputed datasets.

### 2.6 Propensity score matching procedure

To reduce potential confounding caused by baseline imbalance between the non-SICM group and the SICM group, a logistic regression model was used to estimate propensity scores. This model included the following baseline covariates: age, gender, height, and weight. One-to-one nearest neighbor matching (without replacement) was performed using a caliper width of 0.2 times the standard deviation of the propensity score logit.

Standardized mean difference was applied to assess covariate balance after matching, with a threshold of <0.1 defined as adequate balance. The final matched cohort consisting of 478 paired participants (total *n* = 956) was utilized for subsequent model development (Supplementary Table S2).

### 2.7 Statistical analysis

Continuous variables with a normal distribution were reported as mean ± standard deviation, whereas non-normally distributed variables were described as median with interquartile range (IQR). Categorical variables were expressed as frequencies and percentages. Group comparisons for continuous variables were performed using one-way analysis of variance, and categorical variables were compared using the chi-square test. Variables demonstrating significant associations in univariate analyses were subsequently subjected to feature selection using the least absolute shrinkage and selection operator (LASSO) regression. Predictors identified by LASSO were incorporated into a multivariable logistic regression model to construct the final predictive algorithm, which was presented as a nomogram. Model performance was assessed in terms of discrimination (area under the receiver operating characteristic curve, AUC), calibration (calibration curves), and clinical utility (decision curve analysis). Internal validation was conducted using bootstrapping, and external validation was performed with the independent cohort. All statistical tests were two-sided, with a *p*-value < 0.05 considered statistically significant. Analyses were performed using R software (version 4.5.0). This study was reported in accordance with the Transparent Reporting of a multivariable prediction model for Individual Prognosis or Diagnosis (TRIPOD) guidelines.

## 3 Results

### 3.1 Propensity score matching analysis

**Figure 2:**
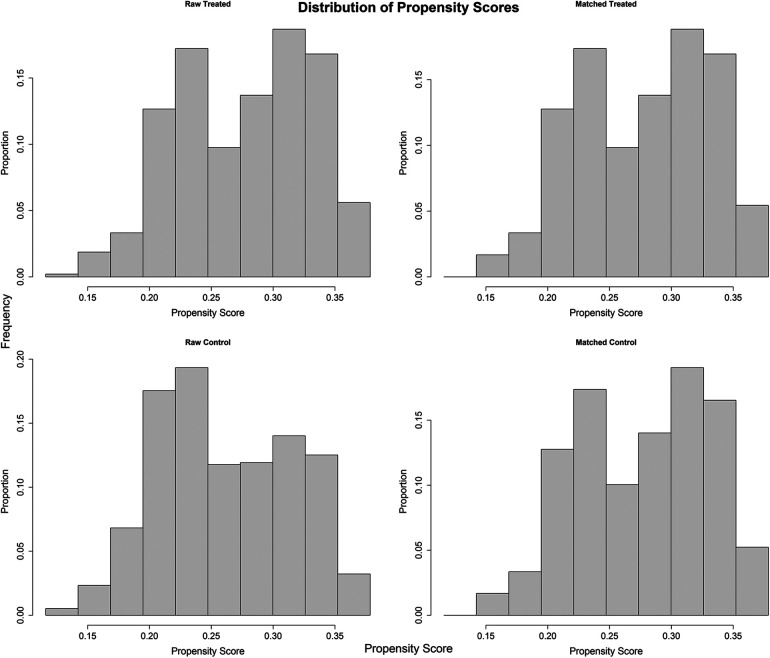
Comparison of baseline characteristic distributions before and after propensity score matching (PSM). The histogram (or density plot) demonstrates the balance of baseline characteristics between the SICM group (treatment/exposed group) and the non-SICM group (control group) before and after PSM. After matching, the distributions in the matched cohort (matched treatment and matched control groups) were more balanced, as evidenced by improved overlap in the histograms (SICM, sepsis-induced cardiomyopathy; PSM, propensity score matching).

A total of 1,814 patients met the eligibility criteria for inclusion. Propensity score matching was conducted using a 1:1 nearest-neighbor matching algorithm without replacement, yielding 478 matched pairs (956 patients) for the final analysis. Prior to matching, a significant difference in sex distribution was observed between the non-SICM and SICM groups (*p* < 0.001). In the non-SICM group, 665 (49.9%) were female and 669 (50.1%) were male, whereas the SICM group comprised 185 (38.4%) females and 297 (61.6%) males. After matching, sex distribution was balanced between the groups: the non-SICM group included 189 (39.5%) females and 289 (60.5%) males, while the SICM group included 183 (38.3%) females and 295 (61.7%) males (*p* = 0.89) (Figure 2). The main characteristics of the derived cohort and the external validation cohort are summarized in Supplementary Table S3. The external validation cohort is smaller in size (*n* = 104 compared to 956), and it comes from a single Chinese center, while the derived cohort comes from a large American database.

### 3.2 Comparison of clinical characteristics

Significant differences in laboratory parameters were observed between the non-SICM and SICM groups. These included phosphate, alanine aminotransferase (ALT), aspartate aminotransferase (AST), lymphocyte percentage, neutrophil percentage, international normalized ratio (INR), prothrombin time (PT), bicarbonate, creatinine, CK-MB, troponin, hematocrit, hemoglobin, mean corpuscular hemoglobin (MCH), mean corpuscular hemoglobin concentration (MCHC), platelet count, red blood cell count (RBC), red cell distribution width (RDW), white blood cell count (WBC), base excess, and lactate (all *p* < 0.05).

Regarding severity scores, significant intergroup differences were noted in APACHE III, Charlson Comorbidity Index, SAPS II, and initial SOFA scores (all *p* < 0.05). Among vital signs, heart rate and respiratory rate differed significantly between groups (both *p* < 0.05). In relation to treatment interventions, invasive mechanical ventilation and the administration of vasoactive agents showed significant differences between the non-SICM and SICM groups (both *p* < 0.05). With respect to comorbid conditions, the prevalence of hypertension also differed significantly between groups (*p* < 0.05). A detailed summary of these findings is presented in Table 1.

**Table 1:** Baseline characteristics of the study cohort stratified by SICM Status.

Variable	ALL (*n* = 956)	Non-SICM (*n* = 478)	SICM (*n* = 478)	*P* value	*SMD*
Demographics					
Age, yr	72.4 (20.5)	72.9 (22.5)	72.2 (18.8)	0.87	0.03
Height, cm	173 (17)	173 (18)	173 (17)	0.52	0.07
Weight, kg	77.3 (26)	77.6 (25.9)	77 (26.9)	0.27	0.08
Male sex, *n* (%)	584 (61.1%)	289 (60.5%)	295 (61.7%)	0.74	0.03
Laboratory parameters within 24 h of ICU admission					
Calcium, mg/dL	8.3 (1.1)	8.3 (1.1)	8.3 (1.1)	0.40	0.10
Chloride, mEq/L	103 (9)	104 (8)	103 (10)	0.12	0.09
Magnesium, mg/dL	2 (0.5)	2 (0.5)	2 (0.5)	0.98	0.03
Potassium, mEq/L	4.2 (1)	4.2 (1.1)	4.3 (1)	0.24	0.07
Sodium, mEq/L	138 (6)	138 (6)	138 (6)	0.73	0.06
Phosphate, mg/dL	4 (2.1)	3.8 (1.9)	4.2 (2.2)	<0.01	0.24
Albumin, g/dL	3 (0.5)	3 (0.5)	3 (0.5)	0.11	0.12
Alkaline phosphatase, U/L	97 (49)	97 (49)	97 (49)	0.27	0.13
ALT, U/L	41 (158)	34 (159)	51 (156)	<0.01	0.08
AST, U/L	59.5 (248.9)	52 (248.9)	71 (247.9)	0.01	0.07
Total bilirubin, g/dL	0.8 (1.2)	0.8 (1.2)	0.8 (1.2)	0.92	0.12
Lymphocyte percentage, %	5.6 (2.4)	5.6 (2.7)	5.6 (2.1)	0.02	0.21
Neutrophil percentage, %	78.7 (7.5)	78 (8.6)	81.9 (10)	<0.01	0.58
APTT, *s*	31.9 (12.3)	31.4 (11.6)	32.45 (12.2)	0.16	0.10
INR	1.3 (0.5)	1.3 (0.6)	1.4 (0.5)	0.01	0.11
PT, *s*	14.7 (5.7)	14.4 (5.3)	15.3 (5.8)	0.01	0.10
Bicarbonate, mEq/L	22 (7)	23 (7)	22 (7)	0.03	0.14
Creatinine, mg/dL	1.5 (1.6)	1.4 (1.6)	1.6 (1.9)	0.01	0.10
CK-MB, ng/mL	6 (12.9)	6 (12.9)	7 (11.9)	<0.01	0.19
Troponin, ng/mL	0.1 (0.3)	0.06 (0.1)	0.19 (0.8)	<0.01	0.53
Hematocrit, %	32.1 (9.3)	31.4 (9.2)	32.8 (8.8)	<0.01	0.20
Hemoglobin, g/dL	10.4 (3.1)	10.4 (3.1)	10.6 (3.1)	0.04	0.14
MCH, pg	30.3 (3.6)	30.6 (3.3)	30.1 (3.9)	<0.01	0.21
MCHC, g/dL	32.64 ± 1.8	32.85 ± 1.9	32.43 ± 1.7	<0.01	0.24
MCV, fL	92 (9)	92 (9)	92 (8.75)	0.53	0.10
Platelet count, ×10^9^/L	196.5 (125.5)	190.5 (120.5)	200 (132.3)	0.03	0.17
RBC count, ×10^12^/L	3.5 (1.1)	3.41 (1.0)	3.53 (1.1)	<0.01	0.23
RDW, %	15.1 (2.8)	14.9 (2.4)	15.3 (3)	0.04	0.15
WBC count, ×10^9^/L	11.2 (7.4)	10.9 (7.1)	11.7 (7.7)	0.04	0
Glucose, mg/dL	135.5 (74.0)	130 (68.8)	140 (80.8)	0.08	0.11
Base excess, mEq/L	−2.5 (5)	−2 (4)	−2.5 (6)	0.01	0.16
Lactate, mmol/L	2.1 (1.3)	2.1 (1.3)	2.2 (1.5)	0.03	0.14
PaCO_2_, mmHg	43 (12.0)	43 (10.8)	43 (12.0)	0.34	0.09
pH	7.33 (0.1)	7.33 (0.1)	7.33 (0.1)	0.29	0.07
PaO_2_, mmHg	104 (75.3)	114 (72.0)	98.5 (72.8)	0.06	0.14
Scores					
APS III	54 (26)	53 (26)	55 (25)	0.01	0.14
Charlson comorbidity index	6 (4–8)	6 (4–8)	7 (4–9)	<0.01	0.30
Initial GCS	15 (15–15)	15 (15–15)	15 (15–15)	0.53	0.02
SAPS II	43 (19.0)	42 (17.8)	45 (19.0)	<0.01	0.18
Initial SOFA	3 (2–5)	3 (2–5)	3 (3–6)	0.01	0.20
Vital signs at 24 h					
Heart rate, beats/min	89 (27)	85 (26)	92 (31)	<0.01	0.61
Mean arterial pressure, mmHg	80.75 (25.0)	80 (25.8)	81 (24.8)	0.95	0.04
Respiratory rate, breaths/min	20 (8)	19 (7)	21 (8)	<0.01	0.25
SpO_2_, %	98 (5)	98 (5)	98 (6)	0.17	0.13
Temperature, °C	36.7 (0.7)	36.7 (0.7)	36.7 (0.8)	0.92	0.01
Interventions within 24 h, *n* (%)					
Invasive mechanical ventilation	118 (12.3)	47 (9.8)	71 (14.9)	0.02	0.15
Vasopressor	154 (16.1)	60 (12.6)	94 (19.7)	<0.01	0.19
Furosemide	301 (31.5)	137 (28.7)	164 (34.3)	0.07	0.12
Analgesia	210 (22.0)	103 (21.6)	107 (22.4)	0.82	0.02
Antibiotics	439 (45.9)	223 (46.7)	216 (45.2)	0.70	0.03
CRRT	126 (13.2)	54 (11.3)	72 (15.1)	0.10	0.11
Comorbidities, *n* (%)					
Hypertension	322 (33.7)	178 (37.2)	144 (30.1)	0.02	0.15
Diabetes mellitus	358 (37.5)	179 (37.5)	179 (37.5)	1	0
COPD	61 (6.4)	33 (6.9)	28 (5.9)	0.60	0.04
Asthma	73 (7.7)	35(7.3)	38(8.0)	0.81	0.02

SICM, sepsis-induced cardiomyopathy; GCS, glasgow coma scale; CRRT, continuous renal-replacement therapy; RDW, red-cell distribution width; MCHC, mean corpuscular hemoglobin concentration; MCH, mean corpuscular hemoglobin; MCV, mean corpuscular volume; ALT, alanine aminotransferase; AST, aspartate aminotransferase; aPTT, activated partial thromboplastin time; INR, international normalized ratio; PT, prothrombin time; PaCO_2_, arterial carbon dioxide tension; PaO_2_, arterial oxygen tension; SpO_2_, pulse oxygen saturation; SAPS, simplified acute physiology score; APS, acute physiology score; SOFA, sequential organ failure assessment; SMD, standardized mean difference.

### 3.3 Variable selection using LASSO regression

**Figure 3:**
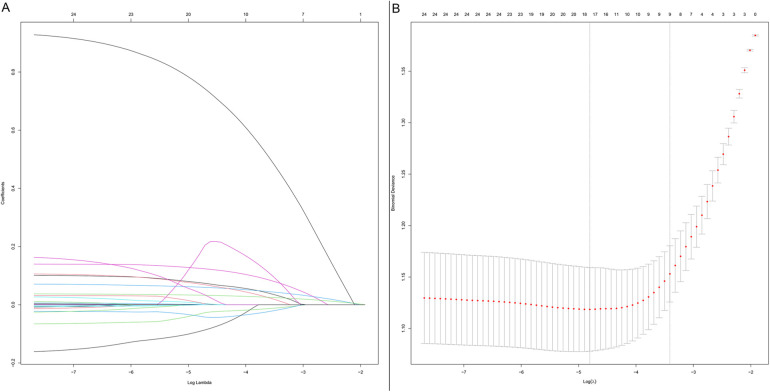
Selection of predictors using LASSO regression. **(A)** Displays the coefficient path plot of the LASSO regression, and **(B)** Presents the results of 10-fold cross-validation for variable selection.

Variables that demonstrated statistical significance in the univariable analysis were incorporated into the least LASSO regression model. With increasing values of the penalty parameter (log *λ*), the coefficients of the predictors progressively approached zero. The optimal value of *λ* was determined through 10-fold cross-validation, resulting in the selection of ten predictive variables: phosphate, neutrophil percentage, troponin, MCH, MCHC, RBC count, Charlson Comorbidity Index, initial SOFA score, and heart rate (Figure 3).

### 3.4 Development of the prediction model via multivariable logistic regression

A multivariable logistic regression model was constructed with SICM as the binary dependent variable (presence = 1, absence = 0) and the LASSO-selected variables as independent predictors. Independent predictors of SICM included phosphate (adjusted OR = 1.122, 95% CI: 1.028–1.227), neutrophil percentage (adjusted OR = 1.072, 95% CI: 1.051–1.094), troponin (adjusted OR = 2.470, 95% CI: 1.923–3.268), RBC count (adjusted OR = 1.431, 95% CI: 1.181–1.741), Charlson Comorbidity Index (adjusted OR = 1.177, 95% CI: 1.110–1.250), initial SOFA score (adjusted OR = 1.094, 95% CI: 1.010–1.186), and heart rate (adjusted OR = 1.038, 95% CI: 1.029–1.048); all with *p* < 0.05 (Table 2).

**Table 2:** Multivariable logistic regression analysis of independent risk factors associated with the development of SICM.

Variable	*β*-Coefficient	Wald_*χ*2	OR	95%CI	*P*-value
Phosphate_icu24h	0.116	617.113	1.122	(1.028–1.227)	0.011
Neu_icu24h	0.069	11,385.390	1.072	(1.051–1.094)	<0.001
Troponin_icu24h	0.904	335.196	2.470	(1.923–3.268)	<0.001
RBC_icu24h	0.358	208.810	1.431	(1.181–1.741)	<0.001
Charlson Comorbidity Index	0.163	1,513.748	1.177	(1.110–1.250)	<0.001
Initial SOFA Score	0.090	714.775	1.094	(1.010–1.186)	0.028
Heart Rate_icu24h	0.038	53,263.323	1.038	(1.029–1.048)	<0.001

SICM, sepsis-induced cardiomyopathy; CI, confidence interval; OR, odds ratio; RBC, red blood cell; SOFA, sequential organ failure assessment.

**Figure 4:**
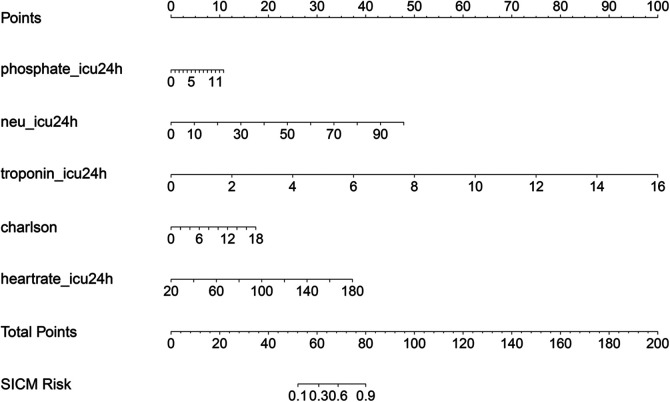
Nomogram for predicting sepsis-induced cardiomyopathy. The nomogram incorporates predictors recorded within 24 h of ICU admission: serum phosphate level (phosphate_icu24h), neutrophil percentage (neu_icu24h), troponin level (troponin_icu24h), heart rate (heartrate_icu24h), and Charlson Comorbidity Index (charlson). SICM, sepsis-induced cardiomyopathy.

For improved clinical applicability and model parsimony, RBC count and initial SOFA score—which contributed less to predictive accuracy—were excluded from the final model. A nomogram was subsequently developed based on the remaining predictors to provide individualized risk estimation for SICM (Figure 4).

### 3.5 Model evaluation and validation

**Figure 5:**
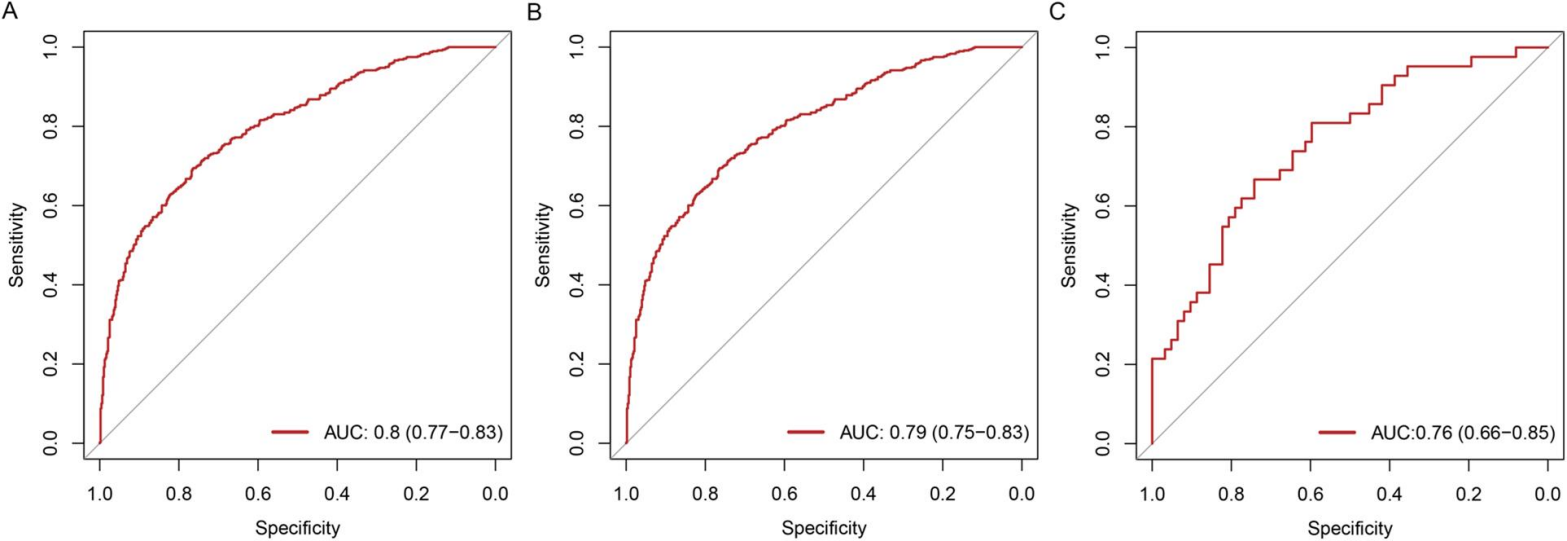
ROC curves for the prediction model. The ROC curves are presented for the **(A)** derivation cohort, **(B)** internal validation cohort, and **(C)** external validation cohort, demonstrating the discriminative performance of the model incorporating independent risk variables.

**Figure 6:**
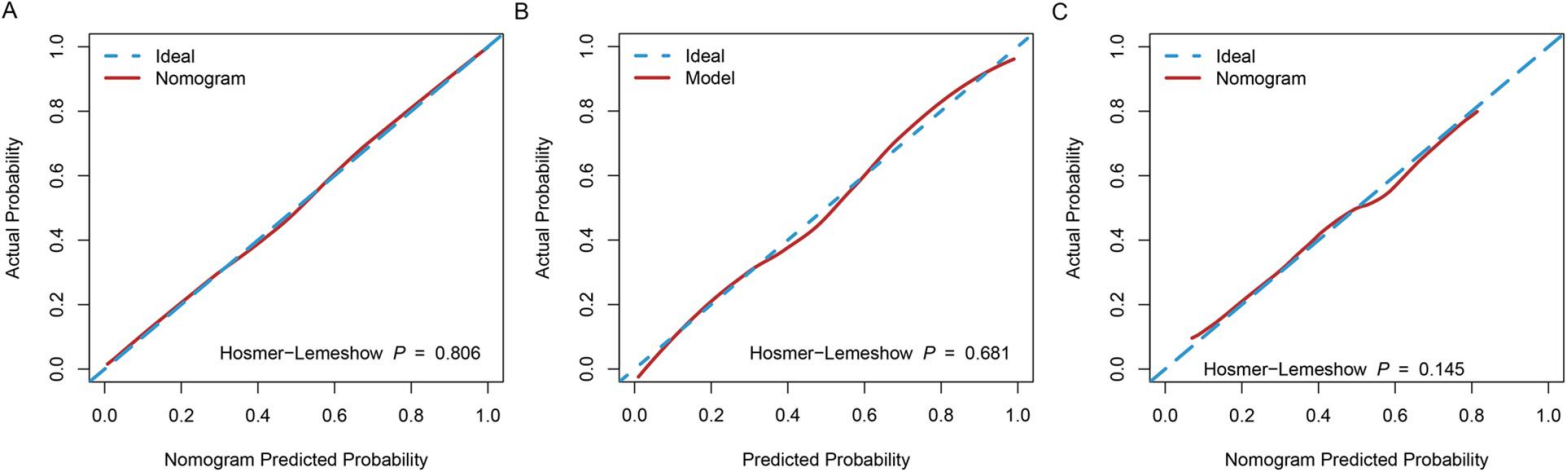
Calibration curves for the prediction model. The plots depict the agreement between predicted probabilities and observed frequencies of sepsis-induced cardiomyopathy in the **(A)** derivation cohort, **(B)** internal validation cohort, and **(C)** external validation cohort.

The prediction model demonstrated strong discriminatory performance in the derivation cohort, with an AUC of 0.80 (95% CI: 0.77–0.83; *p* < 0.001) (Figure 5). Internal validation conducted with 1,000 bootstrap resamples produced a consistent AUC of 0.79 (95% CI: 0.75–0.83). External validation in the independent cohort confirmed robust generalizability, yielding an AUC of 0.76 (95% CI: 0.66–0.85). Goodness-of-fit was evaluated using the Hosmer–Lemeshow test, which demonstrated adequate calibration in the derivation set (*p* = 0.806), the internal validation cohort (*p* = 0.681), and the external validation cohort (*p* = 0.145). Calibration curves further illustrated close agreement between predicted and observed probabilities (Figure 6).

**Figure 7:**
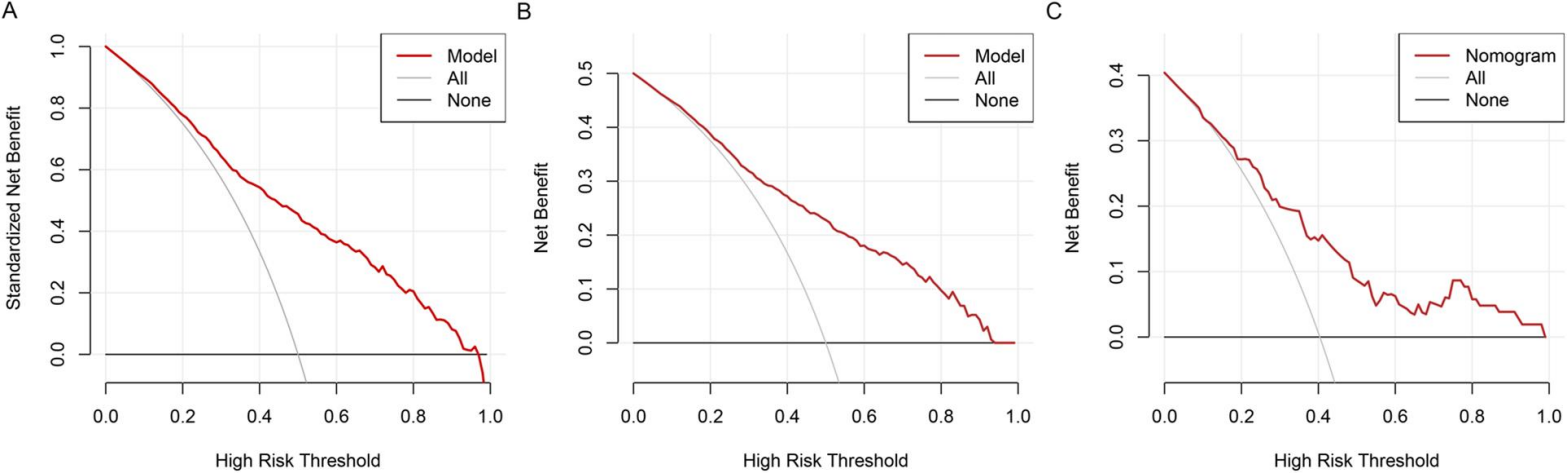
Decision curve analysis for predicting SICM. The net benefit of the prediction model is plotted across a range of threshold probabilities for the **(A)** derivation cohort, **(B)** internal validation cohort, and **(C)** external validation cohort, demonstrating its potential clinical utility.

Decision curve analysis (Figure 7) assesses the clinical applicability of the predictive model by comparing the net benefit of the model with two extreme strategies (“intervene for all patients” and “no intervention at all”). Within a series of threshold probability ranges [i.e., the minimum risk probability of severe infectious complications (SICM) that clinicians consider requiring intervention], the *x*-axis represents the threshold probability, and the *y*-axis represents the net benefit. The gray diagonal line in the figure represents the extreme strategy of “intervene for all patients”, and the horizontal black line (at *y* = 0) represents the extreme strategy of “do not intervene for any patients”. The model curve shows that within the range of threshold probabilities approximately from 0.1 to 0.9, the net benefit obtained by using this model to guide clinical decisions is higher than the two extreme strategies. This indicates that using this model to guide clinical decisions can more accurately identify patients with SICM and avoid unnecessary interventions for low-risk patients. These findings support the potential clinical applicability of this model as a decision support tool for early risk stratification of sepsis.

## 4 Discussion

Sepsis is a life-threatening condition characterized by organ dysfunction secondary to a dysregulated host response to infection (10). The concept of SICM was first reported by Parker et al. in 1984, when biventricular dilatation with reduced left ventricular ejection fraction was observed in a subset of patients with sepsis, presenting clinically as acute heart failure (11). Notably, among survivors, cardiac function frequently showed significant recovery during follow-up.

At present, no universally accepted definition of SICM has been established (9). Several experts, including Martin et al., have characterized SICM as acute cardiac dysfunction in sepsis, occurring in the absence of underlying myocardial ischemia (12).

SICM constitutes a severe complication of sepsis (13). Although some studies have indicated that early screening and timely intervention may improve clinical outcomes, most patients with SICM present with impaired cardiac function at the time of sepsis diagnosis or intensive care unit (ICU) admission. Consequently, management strategies in the ICU have predominantly emphasized hemodynamic stabilization, treatment of associated complications, and evaluation of long-term prognosis and assessment, rather than primary prevention or early detection (14–16).

In this study, analysis of the MIMIC-IV v3.0 database identified five predictors—serum phosphate concentration, neutrophil percentage, troponin level, heart rate, and Charlson Comorbidity Index—for inclusion in the prediction model. Among these, serum phosphate concentration emerged as a particularly significant predictive factor. Previous research has demonstrated that persistently elevated serum phosphate levels are associated with increased short-term mortality in patients with sepsis (17). The present findings further indicate that hyperphosphatemia may contribute to the development of SICM. Elevated phosphate levels during sepsis can promote cellular apoptosis, stimulate inflammatory cytokine release, and increase oxidative stress. These mechanisms may exacerbate disease severity and worsen prognosis (18–20). Additionally, hyperphosphatemia may precipitate arrhythmias or cardiac arrest, potentially facilitating the onset of SICM in patients with sepsis (21).

During the progression of sepsis, inflammatory stimuli induce the mobilization of neutrophils from the bone marrow into the peripheral circulation, resulting in a significant increase in their relative percentage (22, 23). Additionally, pathogen invasion can disrupt neutrophil surface receptor expression, increase the proportion of immature or dysfunctional neutrophil subsets, and inhibit apoptosis, further elevating circulating neutrophil levels (24). As principal mediators of innate immunity, activated neutrophils enhance endothelial adhesion through receptor-mediated mechanisms and release reactive oxygen species, proteases, and vasoactive substances, leading to endothelial injury (25, 26). Subsequently, neutrophils transmigrate across the endothelium into interstitial tissues, exacerbating local myocardial inflammation, increasing microvascular permeability, and promoting tissue edema, collectively reducing cardiac compliance (27).

Multiple inflammatory cytokines are significantly upregulated during sepsis, and dysregulation of these mediators contributes to myocardial injury and dysfunction (28). In the early stages of sepsis, neutrophils rapidly infiltrate cardiac tissue, directly mediating cardiomyocyte damage through the release of inflammatory mediators and reactive oxygen species (29). In the present study, neutrophil percentage was selected as a predictor because it reflects the relative proportion of neutrophils among leukocytes, providing a more accurate representation of systemic inflammatory status than absolute neutrophil counts.

Following myocardial injury, troponin is released from the cytoplasm into the circulation through permeable cell membranes. Sustained injury may also activate proteolytic enzymes, resulting in the dissociation and release of structurally bound troponin (30). As a key biomarker of myocardial injury, elevated troponin levels in patients with sepsis are closely associated with the severity of cardiac dysfunction (31, 32). The mechanisms underlying troponin elevation in sepsis remain incompletely elucidated. Evidence indicates that the primary pathway is not traditional infarction-related cell death but involves systemic inflammation, altered myocardial wall stress, drug-induced cardiotoxicity, and renal impairment (31, 33). Some studies suggest that troponin elevation in sepsis may reflect increased membrane permeability and the release of troponin degradation products rather than direct cardiomyocyte necrosis (34, 35). In the present study, troponin was confirmed as an independent predictor of SICM, with elevated levels indicating cardiomyocyte injury or necrosis and the potential development of cardiac dysfunction in sepsis.

Tachycardia in clinical practice may result from multiple etiologies. In sepsis, systemic stress and heightened sympathetic activation accelerate heart rate by enhancing phase 4 spontaneous depolarization in the sinoatrial node (36, 37). Initially, this tachycardic response functions as a compensatory mechanism to augment cardiac output and maintain tissue perfusion. However, as sepsis progresses and myocardial function declines, persistent tachycardia shortens ventricular diastolic filling time, thereby reducing cardiac output and exacerbating underlying cardiac dysfunction (38). In this study, we utilized the first heart rate measurement recorded within 24 h of ICU admission. This approach reduces potential confounding from subsequent clinical interventions and provides a more accurate reflection of early autonomic nervous system activity and the initial cardiovascular compensatory response, thereby offering valuable predictive insight for the detection of SICM.

The Charlson Comorbidity Index (CCI) is a weighted composite score developed to estimate long-term mortality risk and overall prognosis by accounting for both the number and severity of a patient's comorbid conditions. It provides an objective assessment of baseline health status and cumulative disease burden in patients with SICM. In the present study, higher CCI scores, reflecting greater comorbidity burden and reduced baseline health, were associated with an increased risk of developing SICM and with potentially greater disease severity (39).

To facilitate bedside application, we propose three risk categories based on the predicted probability of SICM derived from the nomogram. Informed by the decision curve analysis (which demonstrated net benefit at threshold probabilities >0.1) and the distribution of predicted risks in the derivation cohort, we suggest the following preliminary thresholds: low risk (<10% predicted probability), intermediate risk (10%–30%), and high risk (>30%). For low-risk patients, routine ICU care without additional cardiac monitoring is reasonable. For intermediate-risk patients, enhanced hemodynamic monitoring (e.g., more frequent heart rate and blood pressure assessments, trending of lactate) and consideration of a targeted echocardiogram if clinically indicated may be beneficial. For high-risk patients, we recommend a formal echocardiographic evaluation within 24 h, optimization of vasoactive support to maintain adequate perfusion without excessive tachycardia, and close follow-up for early signs of cardiac dysfunction. These thresholds are exploratory and should be validated in prospective studies. Local resources and clinical judgment should guide the final decision, as the optimal threshold may vary across different ICU settings.

Although predictive models for SICM have not been widely implemented in clinical practice, several studies have attempted to develop such tools. Sun et al. constructed a predictive model using data from the MIMIC database; however, the study had notable limitations, including a small sample size, exclusion of readily available and potentially predictive variables, lack of propensity score matching to balance baseline characteristics, and absence of external validation (5). Sha et al. (40) employed machine learning algorithms for SICM prediction. While these methods may achieve higher performance than conventional models, they often exhibit “black-box” characteristics, limiting interpretability and potentially leading to overestimation of predictive accuracy.

In contrast, a logistic regression approach offers greater transparency and robustness while maintaining predictive performance comparable to that of machine learning models. Yang et al. focused exclusively on echocardiographic parameters without incorporating additional clinical variables (6), whereas Xiong et al., also using the MIMIC database, included patients with pre-existing coronary artery disease or underlying cardiomyopathy, which may have introduced confounding bias (41).

A primary strength of the present study is the application of propensity score matching to balance baseline characteristics, thereby reducing intergroup confounding. Furthermore, the model was externally validated in an independent cohort, demonstrating strong predictive performance, good generalizability, and potential applicability in clinical settings. Several widely used ICU severity scores, such as SOFA and APACHE, have been validated for predicting mortality and organ dysfunction in sepsis, but they were not specifically designed to identify SICM (5, 6). A direct ROC comparison between our model and these scores was not performed in this study because their validity for the specific outcome of SICM has not been established, and such a comparison could be methodologically problematic. Nevertheless, we acknowledge that a head-to-head comparison in a prospective cohort would be valuable to define the added clinical value of our dedicated SICM model. Future studies are encouraged to address this gap.

The model is designed for bedside use. The five predictors are routinely measured within 24 h of ICU admission, and the nomogram (Figure 4) allows rapid individual risk estimation. For example, a patient with a predicted SICM probability exceeding a clinician-selected threshold (e.g., >20%) might benefit from enhanced hemodynamic monitoring, serial echocardiography, and early consideration of supportive therapies to preserve cardiac function. Conversely, a low-risk patient could be managed with standard care, avoiding unnecessary resource use. The decision curve analysis supports the clinical utility of this approach across a range of threshold probabilities (e.g., >0.1). Prospective implementation studies are needed to confirm whether model-guided management improves patient outcomes.

This study has several limitations. First, as a retrospective analysis, missing data were unavoidable. To mitigate this issue, multiple imputation by chained equations (MICE), a well-validated statistical method for addressing missing values, was employed. Second, several methodological limitations related to echocardiographic assessment should be acknowledged. The timing of LVEF measurement was not standardized but rather dictated by clinical indications, which may introduce selection bias. Although we extracted the first available measurement within a defined time window (72 h of ICU admission), the exact timing varied across patients. Furthermore, LVEF measurements were obtained from routine clinical reports without centralized re-reading or formal assessment of inter-observer variability; thus, measurement error and misclassification are possible. The use of a single LVEF threshold (<50%) as the sole diagnostic criterion for SICM is an oversimplification (42). LVEF is highly dependent on preload and afterload, both of which are frequently and dynamically altered by fluid resuscitation, vasopressor therapy, and mechanical ventilation in septic patients. Consequently, a low LVEF may reflect hypovolemia or reduced afterload rather than true myocardial dysfunction, while a normal LVEF may coexist with significant diastolic or right ventricular dysfunction that is not captured by our definition (43). Future prospective studies should adopt comprehensive echocardiographic protocols—including global longitudinal strain, diastolic parameters, and right ventricular function assessment—with standardized timing and independent core-laboratory interpretation to reduce misclassification risk and more accurately characterize the full spectrum of SICM (44, 45). In addition, as the dataset was derived from the MIMIC-IV database, several clinically relevant and potentially predictive variables, such as procalcitonin, C-reactive protein, and interleukin-6, were not available. The absence of these biomarkers may reduce the comprehensiveness of the prediction model (46). Future studies should consider incorporating these and other dynamic clinical indicators to improve predictive accuracy and enhance clinical relevance. Fourth, the use of propensity score matching before model development, while reducing measured baseline confounding, may have altered the natural distribution of predictors and potentially affects the model's generalizability. To mitigate this concern, we performed external validation in an independent cohort that was not subjected to matching, and the model retained good performance (AUC = 0.76). Nonetheless, readers should interpret the model with caution when applying it to populations with different baseline characteristics. Fifth, our model development strategy combined univariate screening, LASSO regression, and multivariable logistic regression in a sequential manner. While this approach improves clinical interpretability, it may introduce selection bias and optimism in performance estimates. We did not apply post-selection shrinkage methods (e.g., bootstrap shrinkage or ridge penalization), which could lead to overestimation of effect sizes. Future studies should consider using a single penalized regression model (e.g., LASSO without prior screening) or applying shrinkage to the final coefficients to mitigate overfitting. Sixth, the external validation has its own limitations. The sample group for external validation is relatively small (*n* = 104) and comes from a single center, which may limit the general applicability of our research results. As shown in the comparative analysis (Supplementary Table S3), the validation sample group (external validation) and the MIMIC-IV group (MIMIC-IV data) have significant differences in baseline characteristics. Specifically, the serum phosphate level of the validation sample group was significantly lower (median 1.17 milligrams per deciliter compared to 4.00 milligrams per deciliter, *p* < 0.001), the troponin level was higher (median 0.56 compared to 0.10 nanograms per milliliter, *p* < 0.001), the heart rate was faster (median 100 beats per minute compared to 89 beats per minute, *p* < 0.001), the age was also younger (median 65.5 years compared to 72.4 years, *p* < 0.001), and the percentage of neutrophils also had significant differences (78.57% compared to 78.70%, *p* = 0.002). These differences are likely to reflect the differences in the medical system, population characteristics, severity of diseases, and clinical practices between the United States and China. The lower discrimination performance observed in the external validation cohort (derived cohort's AUC was 0.80, while the external validation cohort was 0.76) may be partially attributed to this significant case combination difference, as the predictive factors of the model (such as phosphate, troponin, heart rate) act on different baseline distributions. However, the model still maintained an acceptable discrimination ability in this unique population (AUC was 0.76), indicating that its core predictive relationship is quite robust. Future research should validate this model in larger-scale, multi-center, and more diverse cohorts to confirm its generalizability in different clinical environments and geographical regions. Finally, we did not directly compare the discrimination ability of our model with that of the general severity scores for intensive care units (such as SOFA, APACHE), as these scores were not specifically designed for predicting the severity of myasthenia gravis. Such a comparison would be of great significance for future research, but it is beyond the scope of this current analysis. We acknowledge this as a limitation.

## 5 Conclusion

The SICM prediction model, developed using data from 956 patients in the MIMIC-IV database (v3.0) and externally validated in an independent cohort of 104 patients, demonstrated strong discriminatory performance and robustness. All included predictors are routinely measured clinical parameters, highlighting the model's practical applicability. This tool provides a novel and readily implementable approach for risk stratification, prognostic assessment, and clinical decision support in patients with sepsis.

## Data Availability

The original contributions presented in the study are included in the article/Supplementary Material, further inquiries can be directed to the corresponding author/s.
