# Supplementary material for: Development and validation of a clinical prediction model for sepsis-induced cardiomyopathy
Source: Front Cardiovasc Med. 2026 Jul 17;13:1788162. doi: 10.3389/fcvm.2026.1788162 (PMC13425143; doi:10.3389/fcvm.2026.1788162)
Supplement: Supplementary file 1 [file Table1.docx]

Supplementary Table 1. Variables with >40% Missing Rate

| **Variable** | **Missing rate (%)** |
| --- | --- |
| deathtime_time | 89.12 |
| directbilirubin_icu24h | 93.68 |
| indbilirubin_icu24h | 94.10 |
| tprotein_icu24h | 97.49 |
| CRP_icu24h | 97.21 |
| lymphcount_icu24h | 83.70 |
| neucount_icu24h | 84.02 |
| ddimer_icu24h | 98.39 |
| fib_icu24h | 72.08 |
| uric_icu24h | 96.78 |
| bnp_icu24h | 94.51 |
| Aado2_icu24h | 92.76 |
| last_gcs | 97.11 |
| last_sofa | 78.96 |
| *The candidate variables extracted from the MIMIC-IV database include death time (deathtime_time), direct bilirubin (directbilirubin_icu24h), indirect bilirubin (indbilirubin_icu24h), total protein (tprotein_icu24h), C-reactive protein (CRP_icu24h), absolute lymphocyte count (lymphcount_icu24h), absolute neutrophil count (neucount_icu24h), D-dimer (ddimer_icu24h), fibrinogen (fib_icu24h), uric acid (uric_icu24h), B-type natriuretic peptide (bnp_icu24h), alveolar-arterial oxygen pressure difference (Aado2_icu24h), the last Glasgow Coma Scale score (last_gcs), and the last SOFA score (last_sofa).* | |

**Supplementary** **Table 2**. Comparison of baseline characteristics between the two groups before and after matching

| **Variable** |  | **Before Matching** | | | **After Matching** | | |
| --- | --- | --- | --- | --- | --- | --- | --- |
|  |  | **Non-SCM** | **SCM** | ***P* value** | **Non-SCM** | **SCM** | ***P* value** |
|  |  | **(n = 1332)** | **(n = 482)** |  | **(n = 478)** | **(n = 478)** |  |
| Age (mean (SD)) |  | 70.28 (15.82) | 71.39 (13.94) | 0.17 | 70.96(15.66) | 71.34(13.95) | 0.69 |
| Gender (%) | Female | 665 (49.90) | 185 (38.40) | <0.001 | 189 (39.50) | 183 (38.30) | 0.89 |
|  | Male | 669(50.10) | 297(61.60) |  | 289 (60.50) | 295 (61.70) |  |
| Height (mean (SD)) |  | 172.36 (17.65) | 172.98(15.18) | 0.5 | 172.05(14.91) | 173.08 (15.18) | 0.29 |
| Weight (mean (SD)) |  | 82.57 (24.99) | 80.69(22.13) | 0.15 | 78.82(21.00) | 80.45(21.17) | 0.23 |

**Supplementary Table 3. Baseline characteristics of the derivation and external validation cohorts**

| **Variable** | **MIMIC-IV cohort**  **(n=956)** | **External validation cohort**  **(n=104)** | **P value** |
| --- | --- | --- | --- |
| Phosphate, mg/dL | 4.00 (2.10) | 1.17 (0.56) | <0.001 |
| Neutrophil percentage, % | 78.70 (7.49) | 78.57 (6.98) | 0.002 |
| Troponin, ng/mL | 0.10 (0.29) | 0.56 (0.92) | <0.001 |
| Charlson Comorbidity Index | 6.00 (4.00) | 6.00 (2.00) | 0.452 |
| Heart rate, beats/min | 89.00 (27.00) | 100.00 (19.00) | <0.001 |
| Age, yr | 72.37 (20.49) | 65.50 (27.25) | <0.001 |
| Sex, n (%) |  |  | 0.184 |
| Male | 584 (61.1%) | 56 (53.8%) |  |
| Female | 372 (38.9%) | 48 (46.2%) |  |
| *Note:Values are Mean ± SD or Median (IQR) for continuous variables, and n (%) for categorical variables.* | | | |
